# Syrup of Brown Sugar an Antiseptic

**Published:** 1875-04

**Authors:** 


Syrup of Brown Sugar as an Antiseptic.—I directed its use in an aggravated case of varicella, with entire satisfactory result. The babe, æt. eight months, was thickly pustulated over the head, face, back and loins—was feverish and very restless. To use the language of its mother: "I put the medicine on with a feather thickly over its pustules three or four times, as you directed, and in fifteen minutes my child was fast asleep. She woke up in three hours, feeling free from fever, and was hearty and well." I saw the little sufferer the day before, on the second of March, and it was smiling and cheerful, the angry pustules and inflamed base had disappeared, leaving a flabby ulcer, which dried up. During that and the next day a few pustules appeared on the loins and limbs, which disappeared under repeated application of the syrup.—*Clinic.*

The nearness of the civilized world to the use of uniform weights and measures is shown by the fact that France, Holland, Belgium, Spain, Portugal, Italy, Switzerland, the whole German Empire, and Austria, all use the metric system. Only two great countries stand in the way of its entire and universal adoption—England and the United States. In both of these its use is legal, but not compulsory, and probably they will have to move together in taking any steps.—*Pharmacal Gazette.*

